# Spatio-Temporal Dynamics of Impulse Responses to Figure Motion in Optic Flow Neurons

**DOI:** 10.1371/journal.pone.0126265

**Published:** 2015-05-08

**Authors:** Yu-Jen Lee, H. Olof Jönsson, Karin Nordström

**Affiliations:** Department of Neuroscience, Uppsala University, Box 593, 751 24, Uppsala, Sweden; Lund University, SWEDEN

## Abstract

White noise techniques have been used widely to investigate sensory systems in both vertebrates and invertebrates. White noise stimuli are powerful in their ability to rapidly generate data that help the experimenter decipher the spatio-temporal dynamics of neural and behavioral responses. One type of white noise stimuli, maximal length shift register sequences (m-sequences), have recently become particularly popular for extracting response kernels in insect motion vision. We here use such m-sequences to extract the impulse responses to figure motion in hoverfly lobula plate tangential cells (LPTCs). Figure motion is behaviorally important and many visually guided animals orient towards salient features in the surround. We show that LPTCs respond robustly to figure motion in the receptive field. The impulse response is scaled down in amplitude when the figure size is reduced, but its time course remains unaltered. However, a low contrast stimulus generates a slower response with a significantly longer time-to-peak and half-width. Impulse responses in females have a slower time-to-peak than males, but are otherwise similar. Finally we show that the shapes of the impulse response to a figure and a widefield stimulus are very similar, suggesting that the figure response could be coded by the same input as the widefield response.

## Introduction

White-noise stimuli have been used successfully in visual research in many vertebrates and insects (e.g. [1–3]). White-noise stimuli are powerful in that they relatively rapidly provide the data needed to extract the spatio-temporal response dynamics to a particular stimulus. They have therefore been used to e.g. deduce the directional sensitivity of dragonfly ocellar neurons [4], to record the spatiotemporal dynamics of optic flow sensitive neurons in blowflies [5], and to map the receptive fields of primate retinal neurons [6].

White-noise techniques may treat the relationship between a given input (e.g. a stimulus) and the output (e.g. a biological response) as a time invariant linear system. An ideal linear system shows homogeneity and superposition [7]. Time invariance means that a given stimulus produces an identical response regardless of when it is presented [8], homogeneity that the response doubles in amplitude if the stimulus doubles, and superposition that the response to two consecutive impulses can be predicted from the response to a single impulse.

In some systems it is necessary to introduce terms that account for additional nonlinearities (e.g. [5]), which may be static or dynamic. In other systems, including some biological ones, many underlying nonlinear processes combine in such a way that they produce linear responses within the limits of the experiment (e.g. [9]). Whether linear or non-linear, once the impulse response and the associated non-linearities (if any) of a system are indentified, in theory we can predict how the system will respond to any combination of such impulses, including complex combinations. However, since the impulse responses and non-linearities depend on the statistical properties of the stimulus [5, 10], perfect predictions are rarely possible.

Several different types of white-noise stimuli have been used in insect visual research. Examples include Brownian motion [5], pseudo-random white noise [10], and maximal length shift register sequences (m-sequences) [8, 11]. An m-sequence consists of a string of -1’s and +1’s, specifying the impulses of the stimulus, where +1 corresponds to an impulse of one polarity, and -1 to its opposite. Whereas an m-sequence appears random, it is a deterministic sequence. An m-sequence of the order *n* has a length of 2^*n*^-1, and has the following characteristics [7]: 1) There are 2^*n-1*^ +1’s and 2^*n-1*^-1–1’s; 2) Every string of +1’s and -1’s of length *n* occurs only once; 3) The product of an m-sequence with a time-shifted copy of itself is the same m-sequence, but shifted. Recently, a double m-sequence technique has been used in quantitative fly behavior [12]. In this case two visual stimuli controlled by two independent m-sequences are presented simultaneously, thus allowing the extraction of two impulse responses.

Many animals depend on motion vision cues for navigating in the surround, avoiding obstacles, or visualizing potential prey. One type of biologically important motion is the motion of a figure or feature moving independently of the background. Figure tracking behavior has been particularly well studied in flies and other insects, which orient towards vertically oriented, high-contrast features both during walking [13] and in flight [14]. Human eye movements also orient towards salient features in the visual surround [15], but the behavior is not as robust as fly fixation of vertical bars in the fontal visual field [16].

In the third optic ganglion the fly brain houses lobula plate tangential cells (LPTCs [17]) with large receptive fields. The LPTCs can be broadly divided into a vertical system (VS) and a horizontal system (HS [18]). Whereas most dipteran flies have three HS cells, hoverflies have four [19]. The fourth, hoverfly-unique HS neuron, HS North (HSN), has a remarkably narrow, frontally oriented receptive field [20]. LPTC respond strongly to wide-field motion, but also to local, salient features in the surround. For example, blowfly HS neurons respond strongly to the translation of near-by features in optic flow reconstructed from real flight paths [21–23]. Hoverfly HS neurons have also been shown to respond strongly to salient vertical features within naturalistic panoramas [24].

We here use m-sequences to extract the impulse response of hoverfly HSN neurons to figure motion. We show that the impulse response to a high-contrast, vertical bar maintains its time-course, even when the figure is reduced in size, and that the time-to-peak does not vary significantly across the receptive field. However, we show that the response to a low-contrast figure has a significantly slower time-to-peak and a larger half-width. We further show that the impulse response in female HSN has a longer time-to-peak than in male HSN. Finally, we show that the shapes of the impulse responses to figure and widefield stimuli are similar.

## Materials and Methods

### Animals and electrophysiology

Hoverfly larvae (*Eristalis* spp.) were collected from cow dung at Cederholms Lantbruk. Adult flies were kept in a large net under a 12 h light/dark cycle, at ca. 22°C, and fed with pollen and sugar. At experimental time the hoverfly was immobilized with a bee’s wax and resin mixture. The fly’s head was tilted forward to gain access to the posterior back plate, where a hole was cut over the left lobula plate.

Sharp aluminosilicate electrodes were pulled on a P-1000 Brown-Flaming puller (Sutter Instruments, San Francisco) and filled with 2 M KCl. The electrodes had a typical tip resistance of 50–100 MΩ. The neural signal was amplified using a BA-03X amplifier (npi electronics, Germany) and 50 Hz electrical noise reduced with a HumBug (Quest Scientific, Canada). The signal was acquired at 10 kHz using a NiDAQ 16 bit data acquisition card (NI USB-6210, National Instruments, USA) and the data acquisition toolbox in Matlab (Mathworks, USA). HSN neurons were recorded intracellularly and identified based on their receptive field (Nordström et al., 2008).

### Visual stimuli

The fly was placed 12–13 cm in front of a 160 Hz CRT monitor with a spatial resolution of 640 × 480 pixels, corresponding to ca. 100 × 75° of the hoverfly’s field of view. Visual stimuli were generated using flyfly (http://www.flyfly.se) with the Psychophysics toolbox in Matlab. The stimuli consisted of a 40 pixel wide bar (ca. 6°) or a full-screen background pattern. Both the bar and the background consisted of a random pattern of vertical white and black bars. A new pattern was generated before each trial, using Matlab’s random number generator to get values of 0 (black) or 1 (white). The stimuli were displayed at 100% or 10% contrast.

To quantify the impulse responses to preferred and anti-preferred direction motion in the resting neuron we displayed a stationary full-contrast bar on a mean luminance background. The bar jumped in the preferred or anti-preferred direction with an inter-jump interval of 1.25 s.

All other stimuli were controlled with sequences of non-stationary white noise (m-sequences, see e.g. [7, 25]). Each m-sequence was of the 8^th^ order, thus having a total length of 2^8^–1 (255) impulses. There are 16 possible m-sequence of the 8^th^ order, which can be started at any of the 255 positions. Before each trial we chose one of these from a uniform distribution of pseudorandom numbers, and ran it at 160 Hz. Each m-sequence consisted of a string of -1’s and +1’s, specifying the 1 pixel (ca. 0.2°) velocity impulses of each stimulus in the preferred and anti-preferred direction, respectively.

For the spatio-temporal sensitivity the bar was displayed at 9 azimuthal positions, spaced 25 pixels apart (ca. 4°). Each start position was repeated 1–9 times (4.7 ± 0.6, mean ± std). Before and after each trial the screen was left at mean luminance for a minimum 3 s.

### Data analysis

All data can be found at http://dx.doi.org/10.5061/dryad.dm132. Data were analyzed using Matlab (Mathworks, USA) and Prism (GraphPad Software, USA). We extracted the response kernels [h(t)] using the assumption that the neural response [y(t)] is related to the visual input [given by the m-sequence, x(t)], in the following way:

y(t)=h(t)*x(t)

where “*” indicates convolution and “*t”* time. The impulse response can thus be extracted using circular cross correlation of the neural response and the m-sequence (for step-by-step guides, see e.g. [12]). Here, we used the mathematical definition of circular cross correlation to extract the impulse response in Matlab. Since each experiment used a different m-sequence we first calculated the individual response kernels, before averaging the impulse responses across trials. In each neuron we then quantified the maximum amplitude, the half-width, and the time-to-peak from this impulse response.

For calculation of spatio-temporal sensitivity, bar impulse responses were interpolated across the 9 azimuthal locations using cubic interpolation.

### Validation of the method

To predict the response to a new set of m-sequences we first calculated the average impulse responses for all available data from one neuron except for one trial. We used the averaged impulse response to predict the neural response to the excluded trial, by convolving the impulse response with the excluded trial’s m-sequence. We then calculated the mean percentage error (MPE) by comparing the predicted response and the measured response (as in [26]). To avoid overfitting the data we calculated the optimal length of the impulse response, i.e. the impulse response length that gives the minimal MPE. Return to baseline was calculated as the time it takes for the response to return to, and remain within, 1 standard deviation (sd) of the pre-stimulus membrane potential fluctuations.

We calculated the static non-linearity by comparing the measured neural responses with the predicted responses. Prediction values were first sorted in ascending order and then divided into groups with equal number of data points in each (as in [5]). We then calculated the mean of the prediction values and the mean of the measured responses within each group, through which we fitted a sigmoidal function:

$$y(x)=offset+s/(1+\text{exp}(\frac{\left(\mu -x\right)}{\sigma }).$$

The function was fitted using unconstrained nonlinear optimization. We quantified the impact of the non-linearity on the neural response by re-calculating the MPE after adding the non-linearity.

Throughout the text, n refers to the number of individual trials, and N to the number of animals, where N_HSN_ = N_animals_. All values are given as mean ± sd unless otherwise indicated.

## Results

### Using m-sequences in lobula plate tangential cells

**Fig 1:**
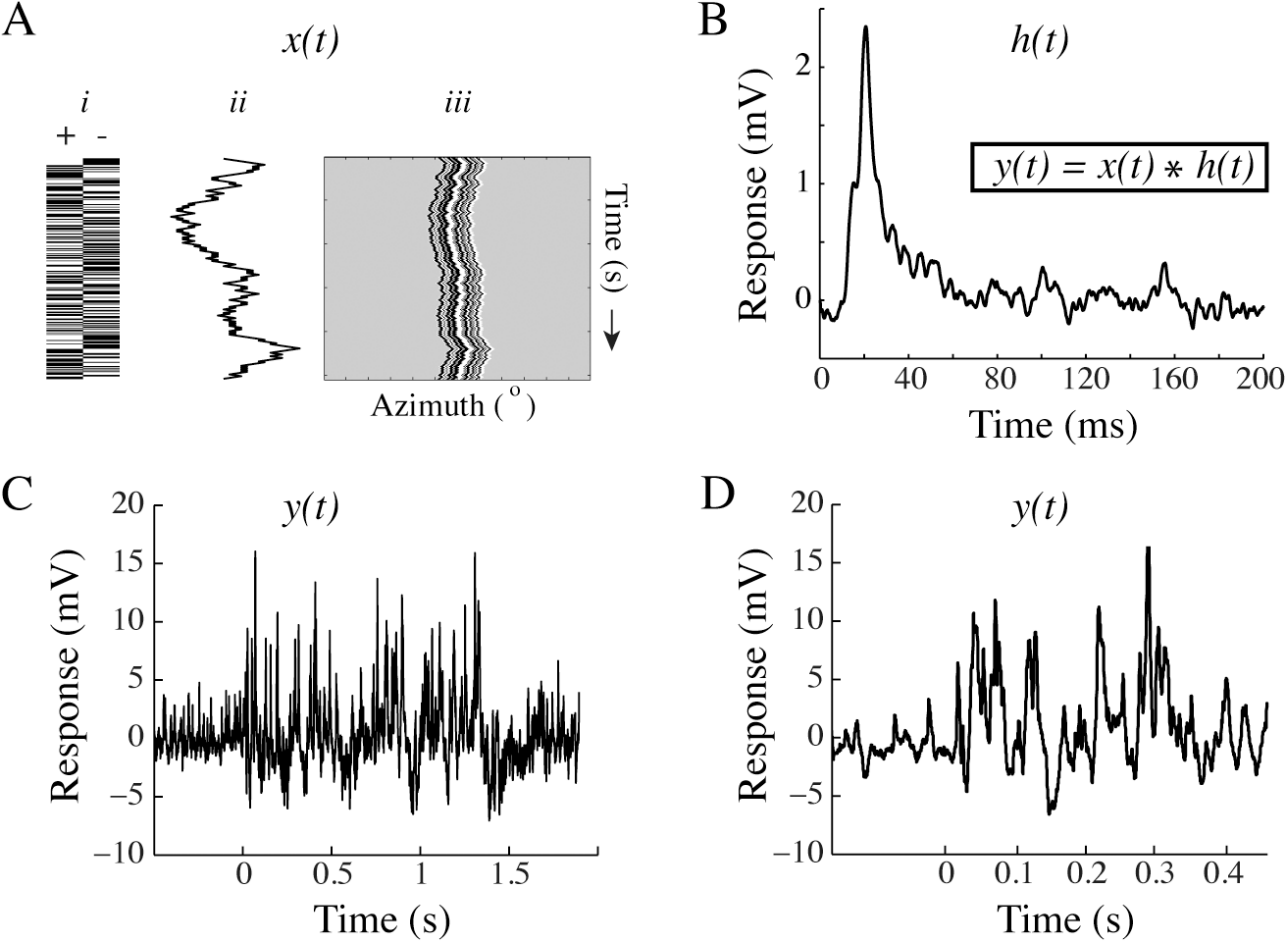
The m-sequence method. A. *i)* Schematic diagram of an m-sequence, consisting of a string of +1’s and -1’s. *ii)* In our experiments, the cumulative sum of the m-sequence describes the relative position of a vertical, striped bar on the screen. *iii)* The resulting space-time plot shows the bar’s motion over time. B. The impulse response kernel extracted from one m-sequence used in one neuron (n = 1). C. The intracellular response of an HS neuron to the m-sequence in panel A. D. Magnification of the data in panel C.

M-sequences can be used to extract the impulse response kernels to visual stimuli [8]. They have recently been used extensively in quantitative behavior (e.g. [9, 27]), but can also be used to measure neural responses [11]. We here use an m-sequence to control the motion of a full-contrast, randomly striped, vertical bar. M-sequences consist of a series of +1’s and -1’s, which are here used to control a figure’s impulse jumps in the neuron’s preferred (left) and anti-preferred direction (right), respectively (Fig 1A *i*). The cumulative sum of the series of impulses in the m-sequence thereby describes the relative position of the bar (Fig 1A *ii*) on the screen over time (Fig 1A *iii*).

White noise techniques describe the relationship between the stimulus input (x(t), the m-sequence) and the output ((y(t), the neural membrane potential) [7]. The impulse response (h(t)), with or without additional nonlinearities, describes the transformation between the two. In the example here, we extract the impulse response (Fig 1B, N = 1) by recording the HSN intracellular response (Fig 1C, n = 1, magnification of the response in Fig 1D) to a stimulus controlled by the m-sequence (Fig 1A). We then deconvolve the response to extract the impulse response corresponding to the bar’s motion. Note that this is the impulse response (Fig 1B) extracted from one trial using a single m-sequence in a single neuron.

### Visual neurons are highly non-linear

**Fig 2:**
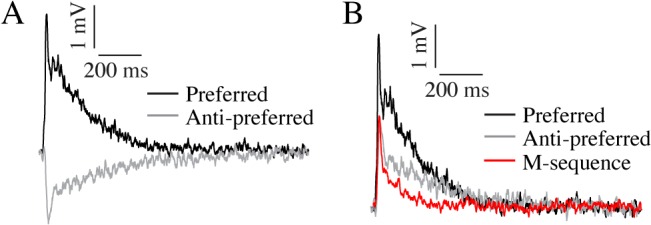
Visual responses are non-linear. A. The impulse response of an un-adapted female HSN to a high-contrast figure jump in the preferred direction (black, N = 1, n = 105) and the anti-preferred direction (grey, n = 126). B. The same data as in panel A, but the anti-preferred direction response is inverted for more direct comparison. The red data show the corresponding impulse response extracted from an m-sequence (from the same female HSN, n = 11).

It is well known that higher-order visual neurons can be highly non-linear (e.g. [28, 29]). To investigate how the non-linearity affects our experiments in more detail, we first identify the velocity impulse response in the resting neuron, where a stationary stimulus performs a single jump in the preferred direction, and then remains stationary in the new position. For this we use the same type of vertical, striped bar as above (6° x 75°, 100% contrast) moving in the center of the receptive field. The response to a preferred direction impulse is characterized by a rapid depolarization, followed by a slower decay to the resting membrane potential (black data, Fig 2A, N = 1, n = 105). The corresponding response to an anti-preferred direction impulse is slower (grey data, Fig 2A, N = 1, n = 126). Across 7 neurons preferred direction time-to-peak is ca 5 ms faster than in the anti-preferred direction (20.2 ± 2.8 ms compared with 25.3 ± 3.9 ms, p<0.05, Student’s paired t-test).

The data in Fig 2A show the impulse responses in a resting neuron. During continuous stimulation visual neurons adapt strongly (e.g. [30]) and impulse responses after adaptation are briefer and smaller than the impulse response in a resting neuron [31]. This is confirmed by our own data where we see that the impulse responses measured in the resting neuron (grey and black data, Fig 2B) have a larger amplitude, and importantly, take longer to return to baseline than the impulse response in the same neuron to an identical bar controlled by an m-sequence (red, Fig 2B, N = 1, n = 11). Across neurons we find that the un-adapted preferred direction impulse has an amplitude of 2.84 ± 0.74 mV, and returns to baseline within 390 ± 96 ms, whereas the anti-preferred direction response has an amplitude of -2.78 ± 0.52 mV, and returns to baseline within 509 ± 205 ms (N = 5 female HSN). This should be compared with the impulse response extracted from m-sequences, which have an amplitude of 2.7 ± 0.3 mV, and return to baseline within 242 ± 25 ms (N = 5 female HSN, p<0.05, Student’s paired t-test).

### Quantification of the non-linearity

**Fig 3:**
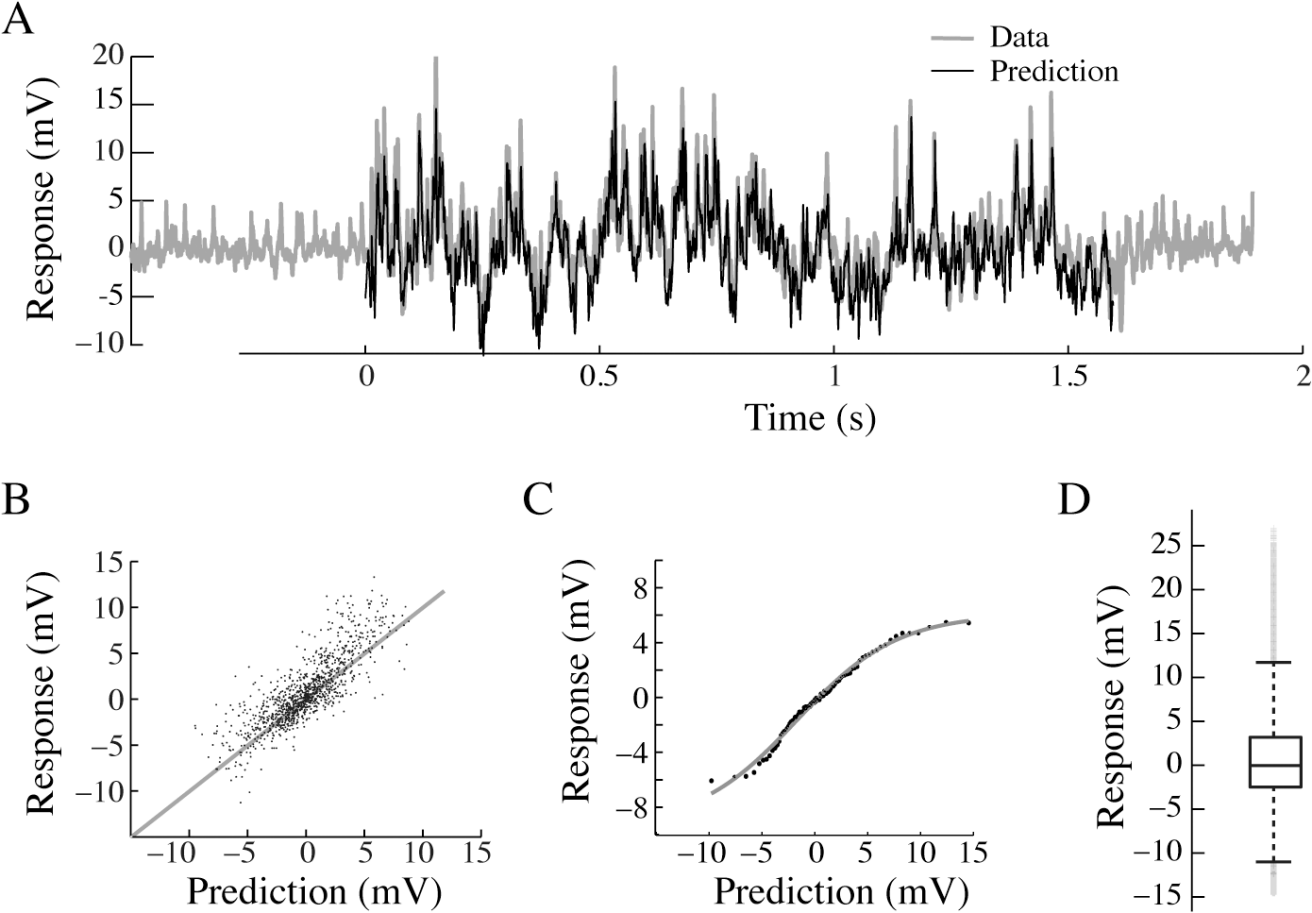
Verifying the use of m-sequences in visual neurons. A. Male HSN response to an m-sequence (grey) and the predicted response (black) based on previously extracted impulse responses from the same neuron. B. The measured response as a function of the predicted response (6 male HSN, MPE = 7.9 ± 14%) to a full-contrast bar in the center of the receptive field. C. The static non-linearity extracted from the same 6 male HSN. The grey line shows the sigmoidal function fitted to the pooled data. D. A box-plot of the membrane potential fluctuations during m-sequence stimulation in the same 6 male HSN. The central mark in the box describes the median, and the edges of the box the 25^th^ and 75^th^ percentiles of the data. The whiskers describe the limits of the data set, excluding the statistical outliers, which are displayed as individual grey data points.

To investigate whether m-sequences can be used at the neuronal level, despite the non-linearities described in Fig 2, we use previously extracted response kernels (as in Fig 1B) to predict the response to a new m-sequence. We do this by convolving the response kernel for the high-contrast figure in the center of the receptive field, with a new m-sequence. Despite the non-linearities described in Fig 2, we find that the predicted response (black data, Fig 3A, n = 1) looks very similar to the measured neural response (grey, Fig 3A, n = 1). To quantify this observation across neurons, we plot the measured response as a function of the predicted response. This analysis shows that the measured response correlates well with the predicted response (Fig 3B, male HSN, N = 6) with a mean percentage error (MPE, [26]) of only 7.9 ± 14%.

The data in Fig 3B suggest that the responses are close to linear across a large part of the response range. However, the most hyperpolarized and depolarized values seem to be overestimated. To account for this non-linearity we quantify it (as described in [5]). Briefly, the data is sorted in ascending order, and sorted in bins with equal number of values in each, through which we fit a sigmoidal function (Fig 3C, male HSN, N = 6). To quantify its impact we re-calculate the MPE with the addition of this non-linearity, and find that it is only slightly improved (5.9 ± 11%; ns, paired t-test). It thus seems as if the non-linearity only plays a minor role in the neural response.

How can the non-linearity of the neural responses be so small? An 8^th^ order m-sequence has a length of 255, and thus contains 128 +1’s and 127–1’s. The number of impulses in the preferred and the anti-preferred direction are thus almost equal [7]. Furthermore, our own data show that in the resting neuron, the preferred direction impulse response is larger than the anti-preferred direction impulse (Fig 2). One would thus expect the neural responses during continuous m-sequence stimulation to be skewed towards depolarizing values. However, a box-plot of the membrane potential fluctuations during m-sequence stimulation shows that the data are symmetrically distributed with the median very close to 0 (Fig 3D, male HSN, N = 6), with only a few statistical outliers that are biased towards depolarizing values (grey data points, Fig 3D). It thus seems as if the neuron is only depolarized for brief moments (see raw data in Fig 3A), and spends longer durations at hyperpolarized values, which would make the data more evenly distributed (Fig 3D) than predicted. The predominantly symmetrical distribution might contribute to the small static non-linearity (Fig 3C).

### The impulse responses to smaller figures have similar time-course

**Fig 4:**
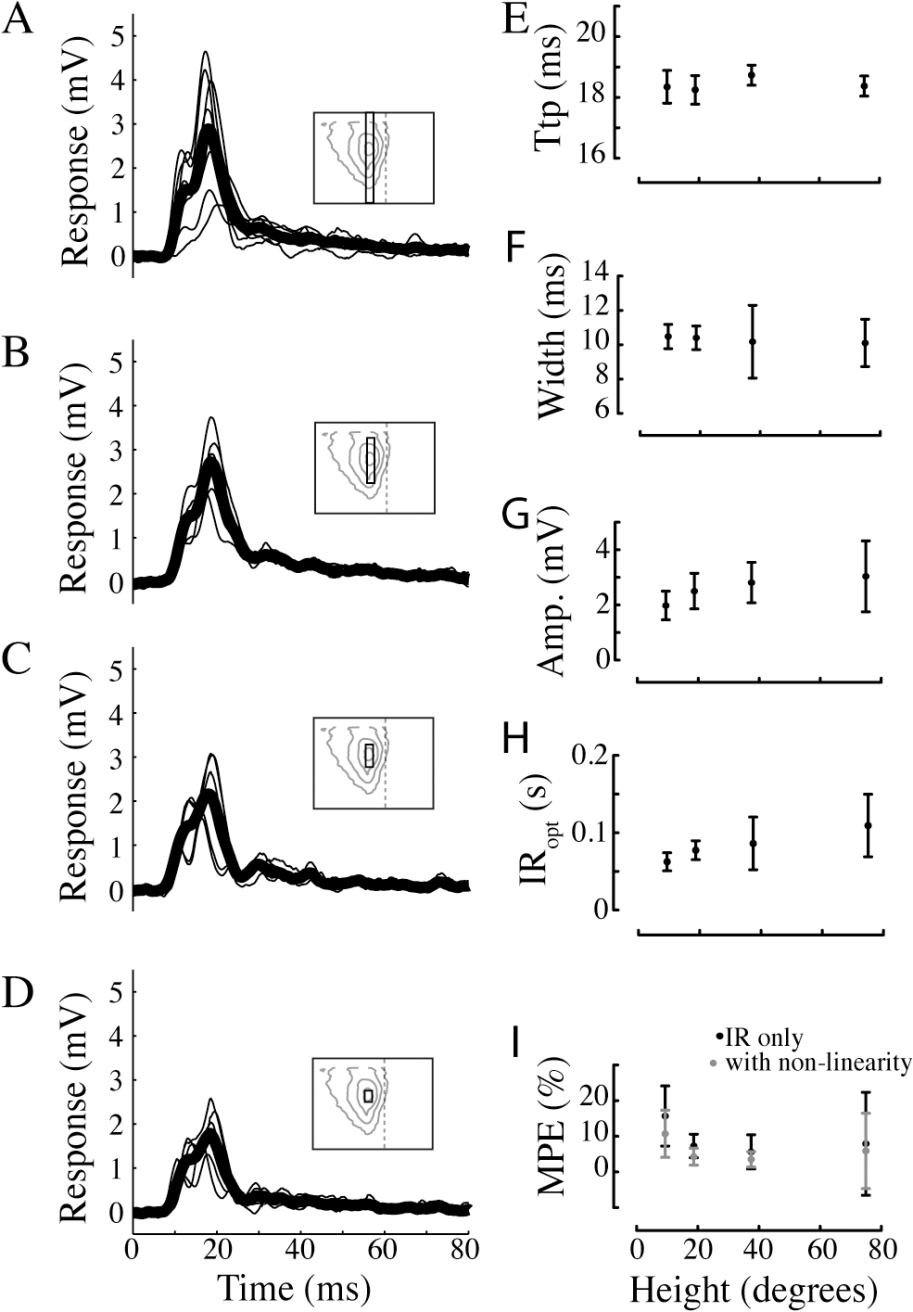
The impulse response to different figure sizes. A. The impulse response to full-contrast bar moving over a grey background. The bar covered the vertical extent of the screen (75°, see inset pictogram for the bar’s height relative to the HSN receptive field as projected on the visual display). The individual impulse responses from each neuron are displayed with thin lines, and the average impulse response across neurons is displayed with the fat black line (N = 7). B. The impulse response to a 37° high bar (N = 5). C. The impulse response to a 19° high bar (N = 5). D. The impulse response to a 9° high bar (N = 5). E. Time-to-peak (Ttp) values for the data in panels A-D. F. Half-width values, i.e. the width of the impulse response at 50% maximum amplitude, for the data in panels A-D. G. Peak amplitude values, i.e. the maximum amplitude, for the data in panels A-D. H. The optimal impulse response (IR_opt_) length for the data in panels A-D, defined as the impulse response length that gives the minimal MPE. I. The minimum MPE for the data in panels A-D. The black data show the results using only the impulse response, and the black data the MPE after adding the static non-linearity. In panels F-I the data show mean ± sd.

We next measure how the figure response is affected by the height of the bar. The impulse response to a full-screen bar moving in the center of the receptive field, over a mean-luminance background (Fig 4A, N = 7) has a time-to-peak of 18 ms (Fig 4E) and a half-width of 10 ms (Fig 4F). When the bar is reduced to a height of 37° (Fig 4B, N = 5) the impulse response amplitude (Fig 4H) and time course (Fig 4F and 4G) remain virtually identical. When the bar height is reduced further, to 19° (Fig 4C, N = 5) or 9° (Fig 4D, N = 5) the response amplitude is reduced, but not significantly (Fig 4H, one-way ANOVA). The time-to-peak and the half-width of the impulse responses remain unaltered (Fig 4F and 4G; ns, one-way ANOVA).

In all cases we quantify the MPE of impulse responses of different lengths to identify the optimal impulse response length, which reduces the MPE maximally without overfitting the data. For the experiments described here the optimal impulse response length is about 84 ms (Fig 4H), with no significant difference between figure heights. The MPE at the optimal impulse response length is 9.1% (black data, Fig 4I). To quantify the impact of the non-linearity, we recalculate the MPE after its addition (grey data, Fig 4I). This analysis shows that the non-linearity reduces the MPE from an average 9.1% to 6.7%, but the reduction is not significant (2-way ANOVA), confirming our previous observation (Fig 3) that the static non-linearity does not play a major role in the neural response to m-sequences.

### Spatio-temporal response profile

**Fig 5:**
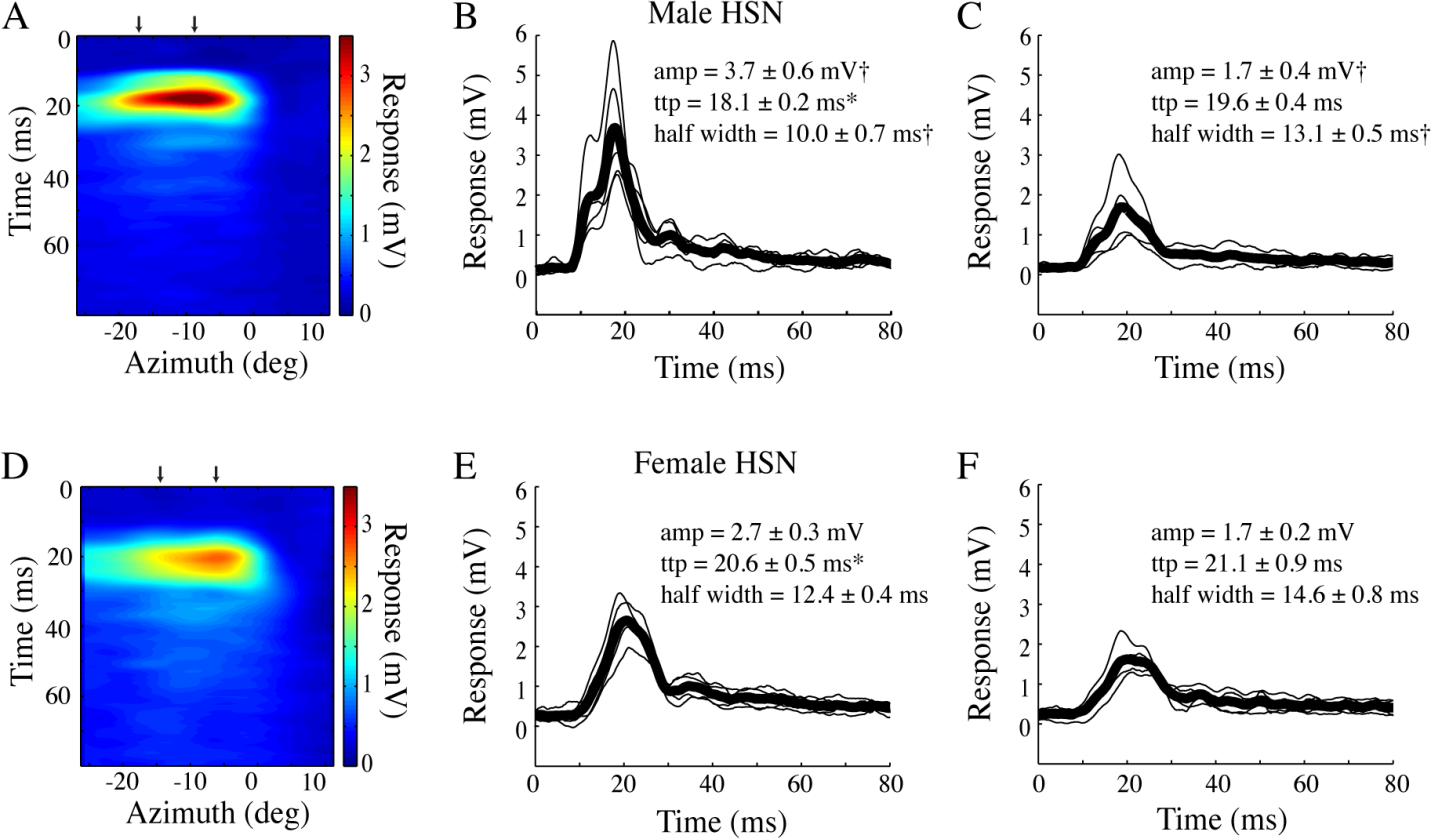
The spatio-temporal profile of the bar impulse response. A. The male HSN spatio-temporal impulse response profile shows the bar’s azimuthal position on the x-axis (interpolated from 9 start positions), the temporal profile of the impulse response on the y-axis, with the response amplitude color-coded. The bar moved on a grey background. N = 5. B. The individual impulse responses from the center of the receptive field (see arrow in panel A) in thin black, and the mean in fat black line. C. The impulse responses from the peripheral receptive field (see arrow in panel A). D. The spatio-temporal profile for female HSN. N = 4. E. The individual impulse responses from the center of the female HSN receptive field in thin black, and the mean in fat black line. F. The impulse responses from the female HSN peripheral receptive field. In panels B, C, E and F stars (*) indicate significant differences between the sexes, whereas crosses (†) indicate significant difference between stimulus locations (central or peripheral receptive field). Significance (p<0.05) was tested with one-way ANOVA followed by Bonferroni's multiple comparison tests.

To investigate the spatial profile of the response, we display the bar at different positions across the azimuth and interpolate the individual impulse response kernels. The spatial distribution of the figure response (Fig 5A, N = 5) largely reflects the underlying male HSN receptive field [20]. Compared with the impulse response through the center of the receptive field (Fig 5B), the impulse response in the periphery (Fig 5C), is smaller in amplitude and has a broader half-width (p<0.05, one-way ANOVA followed by Bonferroni's multiple comparison test). However, there is no significant difference in time-to-peak between the central and peripheral receptive field.

In female HSN (Fig 5D, N = 4) we find a slightly broader spatial profile, consistent with earlier work showing that female hoverflies have larger HSN receptive fields [20, 32]. The impulse response through the center of the receptive field (Fig 5E) has a slightly longer time-to-peak than the impulse response through the center of the male HSN receptive field (p<0.05, one-way ANOVA followed by Bonferroni's multiple comparison test). The impulse response in the female peripheral receptive field has a similar time-to-peak as in the central receptive field, and a slightly broader half-width (but not significant, p>0.05, one-way ANOVA followed by Bonferroni's multiple comparison test).

### The impulse responses to low contrast stimuli are slower

**Fig 6:**
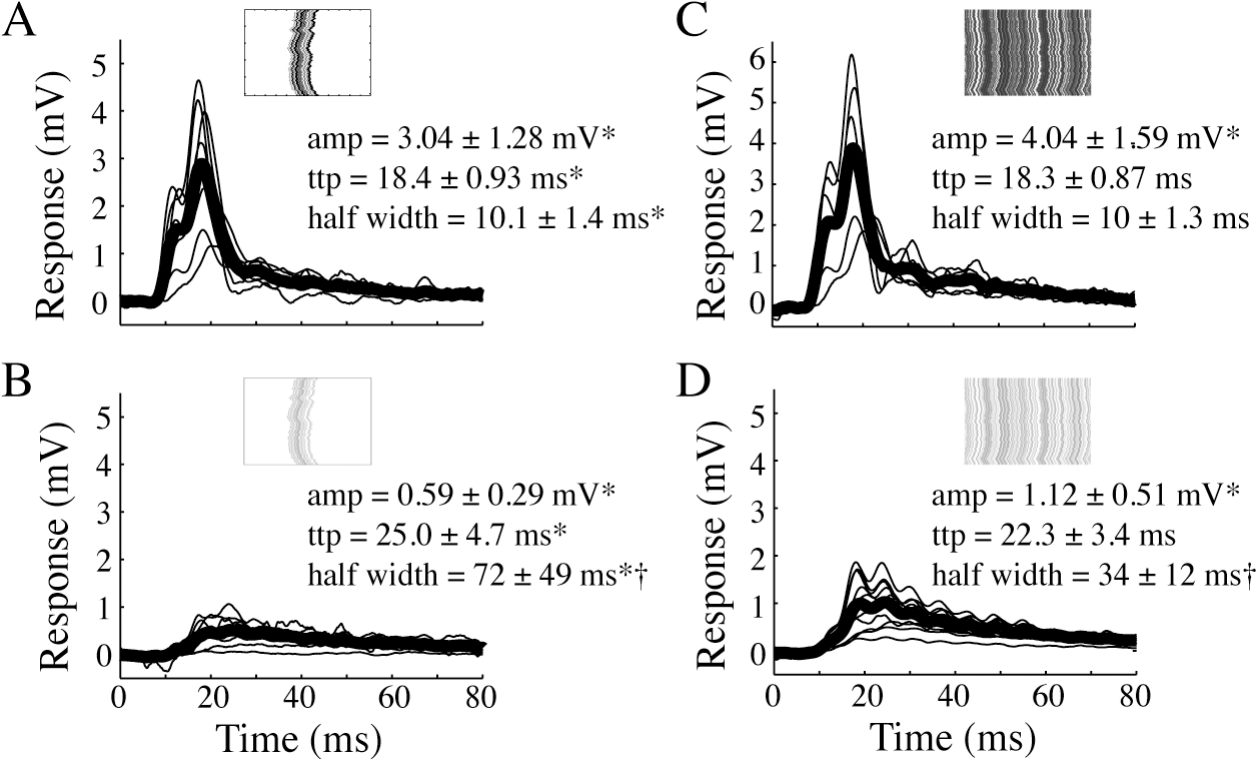
The impulse response to low contrast stimuli is slower. A. The impulse response to a full contrast vertical bar (male HSN, N = 8). B. The impulse response to a 10% contrast bar (male HSN, N = 11). C. The impulse response to a full contrast, full screen background pattern (male HSN, N = 7). D. The impulse response to a 10% contrast, full screen background pattern (male HSN, N = 12). In all panels the pictograms display example space-time plots. Stars (*) indicate significant differences between contrast conditions, and crosses (†) indicate significant differences between stimulus types (bar or background). Significance (p<0.05) was tested with one-way ANOVA followed by Bonferroni's multiple comparison tests.

The time-to-peak of the male HSN impulse responses described above remained similar across conditions where the response amplitude was reduced by changing the height of the bar (Fig 4) or its placement in the visual field (Fig 5). We next investigate what happens to the impulse response when the contrast of the figure is rescaled to 10%. As expected, the impulse response to a low-contrast bar (Fig 6B, N = 5) has a significantly smaller amplitude than the impulse response to the high-contrast bar (Fig 6A, N = 7; p<0.05, one-way ANOVA followed by Bonferroni's multiple comparison test). The impulse response to the low contrast figure also has a slower time course with a significantly longer time-to-peak and wider half-width (Fig 6B).

LPTCs are traditionally believed to be tuned to widefield movement stimuli [33]. Are the HSN impulse responses to figure motion different from the impulse responses to widefield motion? We find that the impulse response to the motion of a 100% contrast background pattern (Fig 6C, N = 12) has similar time-to-peak and half-width as the impulse response to a full-contrast figure (Fig 6A), but a slightly larger amplitude. However, the amplitude does not scale up 16 times, despite the full-screen stimulus being 16 times larger than the bar (see [34] for a thorough description of dendritic gain control). When the background contrast is rescaled to 10% (Fig 6D, N = 11), the amplitude is significantly reduced compared with the full-contrast background response (p<0.05, one-way ANOVA followed by Bonferroni's multiple comparison test). The time-to-peak and half-width are more similar to the 10% contrast figure impulse response (Fig 6B) than to the full-contrast background impulse response (Fig 6C). The similar impulse responses to figure and background motion, whether displayed at full-contrast or reduced contrast, suggest that the figure response could be driven by similar underlying input dynamics.

## Discussion

### White noise techniques

White-noise techniques have been used extensively for investigating neural responses to different visual stimuli. The impulse responses that we extract are faster than some of the insect impulse responses measured previously. For example, the time-to-peak of the impulse response in the locust lamina is ca. 40 ms [35], in blowfly medulla amacrine cells ca. 40 ms [36], in *Drosophila* medulla neurons ca. 50 ms [37], and in the blowfly LPTC H1 ca. 50 ms [38]. However, direct comparisons are difficult since the experiments differ in terms of species, recording conditions and stimulus techniques. These factors all affect the response dynamics of neurons in the visual system. For example, the temperature [39] and the levels of stress hormones in the fly brain [40] have a strong effect on the response latency of visual neurons. It is also well known that parameters of the visual stimulus, such as its temporal frequency and contrast, have a large effect on the neural response onset [41]. Therefore, the fact that the blowfly H1 recordings were performed in very dim light [38] could explain the slower responses. Indeed, *Drosophila* Foma-1 neurons, which are also located in the lobula complex, have a time-to-peak of 20 ms [42], more similar to the responses measured here. However, since the lamina and medulla are presynaptic to the lobula plate, one would expect those responses to be faster than those we measured in HSN. This remaining discrepancy might be explained by recent work which showed that *Eristalis* visual responses can be as much as 10 ms faster than *Calliphora* responses to the same stimuli [43].

The high similarity between the predicted responses to novel stimuli, and the measured HSN response to the same stimulus, show that the m-sequence method used here is robust and reliable (Fig 3A and 3B). We found MPEs around 9% for the linear model and around 6% after adding the static non-linearity (Fig 4I). It is intriguing that the addition of static non-linearities only slightly (and not significantly) improved the prediction of responses in our neurons given that previous studies have shown that the insect visual can display significant nonlinearities (e.g. [10, 44]). However, static non-linearities in lamina neurons and LPTCs have been shown to vary substantially between neuron types and pathways (e.g. [5, 37]). For example, the static non-linearity in the L2 pathway is much stronger that the static non-linearity in the L1 pathway [37]. In experiments comparing the blowfly LPTCs H1 and V1, the static non-linearity was larger in H1 [5], despite these neurons otherwise being physiologically similar.

In fly photoreceptors linear models have been found to be poor predictors of visual responses, with MPEs around 25% [26, 45]. These errors were not due to noise as they were significantly reduced to values comparable to those found here in LPTCs when non-linearities were added. In L-neurons from the dragonfly ocelli, linear predictors produced MPEs as low as 10% when using optimal stimuli [10]. In a recent study the spatio-temporal receptive fields of LPTCs were determined using Brownian motion [5], with predictive powers between 0.3 and 0.7. Predictive power was defined as the component of the response that could be explained by a model describing the impulse response and the receptive field. In quantitative behavior it has been shown that purely linear models predict the behavior well [12], at least in the frontal visual field [27]. It is thus tempting to suggest that linear models become more useful for predicting responses the higher we travel up the visual processing pathway.

Previous work has shown a strong difference between adapted and un-adapted responses in HS neurons, as we saw in Fig 2. For example, the impulse response in the adapted neuron has a smaller amplitude and a briefer time course compared with the un-adapted response [31]. The reduced response amplitude following motion adaptation is thoroughly established, and has been well investigated (see e.g. [46–48]). It is likely that the altered response magnitude, and time course, following adaptation serves a role in allowing neurons to appropriately respond to novel stimuli [49–51].

### Lobula plate tangential cells as figure detectors

The physiological response properties of LPTCs correlate well with the behavioral optomotor response, and they are therefore generally described as underlying the optomotor response. However, the motion of local, high-contrast features also influence the LPTC responses. For example, during optic flow reconstructed from real flight paths, the translation of near-by, high-contrast features generates strong transient responses in blowfly HS neurons [21–23]. Indeed, the influence of the bar’s motion on the HS neurons’ membrane potential fluctuations was in some cases stronger than the influence of the background motion [52]. The response to individual features within naturalistic widefield optic flow is called pattern noise [24]. In blowflies, the pattern noise response to the relative motion of a vertical object is particularly strong if the background is simulated to be distant [48]. The pattern noise depends on the receptive field, so that the hoverfly HSN, which has a much smaller receptive field than HSNE [20], displays stronger pattern noise [24], particularly to vertically elongated features.

Behavioral experiments on *Drosophila* show that the bar fixation response is strongest in the frontal visual field [25, 53], coinciding with the peak sensitivity of the hoverfly HSN receptive field [20]. Interestingly, *Drosophila* track bars consisting of higher-order motion cues, not coded by typical delay-and correlate-type input [25, 54]. We recently found that hoverfly HS neurons respond to similar bars consisting of higher-order motion cues [55], with particularly high sensitivity in the frontal visual field [55]. In this context it is interesting to note HS neurons were originally suggested to be involved in bar fixation behaviors (see e.g. [17]). However, even if a neuron responds to a stimulus, this does not imply that it was ‘designed’ for this purpose. Therefore, it is interesting that the impulse responses to figure motion (Fig 6A and 6B) are remarkably similar to the impulse responses to background motion (Fig 6C and 6D), suggesting the possibility for similar underlying coding. Furthermore, recent work has shown that the asymmetrical neural responses to preferred and anti-preferred direction motion (as seen here in Fig 2) could contribute to the observed sensitivity to figure motion in LPTCs [56].

### Behavioral impulse responses to figure motion

Behavioral experiments using m-sequences in *Drosophila* show figure responses that differ remarkably from behavioral background motion responses [27]. Indeed, the figure responses extracted from behavior have a sustained component with a very slow return to baseline, more similar to a step response than to an impulse response. This is very different from the neural impulse responses to figure motion that we describe here (Fig 4). Furthermore, the figure responses recorded in *Drosophila* behavior depend substantially on where in the receptive field the figure is presented, so that only figures in the frontal visual field are tracked, whereas those in the rear visual field lead to reduced tracking [27]. We could only record figure responses in the frontal visual field (Fig 5), and not in the dorsal visual field, since this is outside the HSN receptive field [20]. Nevertheless, the strikingly different responses to figure motion in *Drosophila* behavior and *Eristalis* LPTCs suggest that behavioral figure tracking may be supported by a different neural pathway (see e.g. [57]).

The impulse responses to background motion recorded in *Drosophila* behavior [27] look more like the LPTC background responses we recorded here (Fig 6). However, a more direct comparison of *Drosophila* HS and behavioral impulse responses to widefield motion show that whereas the neural response quickly returns to baseline, the behavioral response remains significantly elevated for several seconds [11]. This suggests that the HS response does not correspond directly to the behavioral optomotor response, but that there is a leaky integrator between the HS cells and the motor output [11].

### Species considerations

Importantly, whereas the lamina and medulla neurons are highly conserved across flies [58], there is much more variation in the lobula and lobula plate [19]. Whereas *Calliphora* [59], *Musca* [60] and *Drosophila* [61] have three HS cells in each hemisphere, *Eristalis* hoverflies have four [20]. Compared with the other dipteran HS cells, the hoverfly HSN is unique with a very narrow, frontally oriented receptive field [20]. *Calliphora* and *Drosophila* HS cells have receptive fields that extend laterally, with peak sensitivity in the lateral visual field [59, 61], which makes more sense for neurons tuned to the motion of yaw optic flow [62]. However, despite the small receptive field, *Eristalis* HSN responses are strongly correlated with yaw motion [20].

Besides the neural differences, compared with the more commonly studied dipteran flies, *Eristalis* hoverflies perform elaborate flight behavior. In highly cluttered environments male hoverflies set up and vigorously defend their territories, from which they pursue intruding conspecifics [63]. *Eristalis* pursuits are extremely precise, high-speed and advanced, and they have been shown to intercept the target [64]. In contrast, even if male *Fannia* [65], *Calliphora* [66] and *Musca* [67] follow moving targets, they do this at lower speed and they use a smooth-pursuit mode. Furthermore, compared with *Calliphora* the free flight patterns of *Eristalis* are also more elaborate, with stationary hovering, backwards and sidewards flights, and extremely rapid turns [68]. Indeed, *Eristalis* hoverflies reach velocities of 10 m/s, and can turn at many thousand degrees per second [64], compared with 1–2 m/s and a few thousand degrees per second in *Calliphora* [22] and *Fannia* [69]. It is thus potentially possible that the high sensitivity to bar motion that we have described here reflects the more elaborate flight behavior of hoverflies, rather than something universal for dipteran LPTCs.
